# Supplementary material for: Zipf’s law in China’s local government work reports: A 21-year study using natural language processing and regression analysis
Source: PLoS One. 2025 May 20;20(5):e0324713. doi: 10.1371/journal.pone.0324713 (PMC12091883; doi:10.1371/journal.pone.0324713)
Supplement: Attachment — Submitted filename: Response to Reviewers.doc [file pone.0324713.s004.doc]

Dear Dr. Pruller and Reviewers,

Thank you for your insightful comments and suggestions on our manuscript, “Zipf’s Law in China’s Local Government Work Reports: A 21-Year Study Using Natural Language Processing and Regression Analysis”. We really appreciate the time and effort dedicated to evaluating the work and find the feedback invaluable in strengthening the manuscript. We have carefully addressed each point raised and believe the revisions significantly enhance the study’s rigor, clarity, and overall impact. All changes have been highlighted in red in the manuscript in response to the reviewers’ comments, and a list of the main modifications include:

•We have revised the abstract, introduction and literature review.

•We have refined the methodological details by providing specific information on the Jieba word segmentation tool, the implementation of a double-logarithmic model, and the development of a custom dictionary. Furthermore, we have enhanced the analysis of our findings, with a particular emphasis on the substantial inter-regional and inter-temporal variations observed in the Pareto coefficients.

•We have included detailed descriptive statistics of the length of government work reports,which is now in table 1.

•We have made the limitations and future research directions more concrete in the conclusion section. Additionally, we have removed unsubstantiated claims about practical implications.

In the subsequent sections, we have included a detailed response to the feedback from both reviewers. The reviewers’ comments are marked in blue, while our replies are presented in black.

Sincerely,

Yanfang LI

**Specific Responses to Reviewer Comments:**

We are immensely grateful to the reviewers for their invaluable feedback and constructive suggestions, which have been meticulously incorporated into our revised submission. We believed that that revised one has greatly improved. Below, you will find our detailed explanations and responses to each of your recommendations.

**Reviewer #1:**

Although variations in Pareto coefficients (key to measuring this relationship) exist, they tend to values close to 1, indicating linguistic patterns consistent with Zipf’s Law. However, quantitative results of analysis and inference are necessary to provide strong evidence for the presence of the Law. At a minimum, a descriptive analysis that helps readers understand the strength of the empirical evidence, as well as the specific deficiencies noted in the text, is required. In particular, the heterogeneity across regions:

- Some provinces showed significant differences in Pareto coefficients. For instance, Guizhou had the highest average, while Hunan recorded the lowest.

- These differences reflect variations in priority topics and linguistic styles due to regional or political factors.

**Authors:** We have expanded the discussion of the Pareto coefficients, providing a more descriptive analysis of their distribution(see table1and table2).We have also explicitly addressed the heterogeneity across regions by analyzing the variations in Pareto coefficients in relation to historical, regional, and political factors, including the impact of the National Congress of the Communist Party of China. The discussion now provides more nuanced interpretations of these regional differences and their potential causes.

A clear presentation of data visualizations would help readers frame the author’s claims within an empirical evidence context. In general, the methodological details are sparse. To strengthen the article’s credibility, additional details enabling result replication would be beneficial. In summary, the paper mentions using Zipf’s Law to analyze predominant words and topics.

**Authors:** We have included a histogram (Fig3) that shows the distribution of Pareto coefficients, providing a clear visual representation of our findings. Additionally, we have included a scatter plot (Fig2) to show the overall dispersion of the Pareto Coefficients.

Furthermore, we have substantially expanded our description of the methodological process, including providing details of our custom Jieba dictionary and detailing the steps that we have taken during data pre-processing. We have also now included a comprehensive explanation of the double logarithmic regression model and why we only used word forms and not lemmas in the article. Additionally, the technical roadmap (Fig1) shows all the steps.

**Reviewer #2:**

Abstract

0 - Rewrite after review. The abstract is concise but lacks specificity about the theoretical implications, practical consequences and limitations of the study. The authors should clearly state whether their findings confirm or challenge existing hypotheses about Zipf’s law in structured political discourse. Additionally, the abstract could benefit from a brief mention of methodological limitations, such as potential biases in word segmentation and data preprocessing.

**Authors:** We have revised the abstract to address your feedback, adding specificity regarding theoretical implications, practical consequences, and limitations.

1- Authors claim that (lines 15-18): “Our findings have practical implications for enhancing government report clarity and transparency by adhering to linguistic patterns that align with Zipf’s law. This study offers a valuable framework for gaining deeper insights into the linguistic characteristics of local government work reports in China”. Please review this big claim, and explain better what are that “practical implications” and what suppose that for government reports in China.

**Authors:** We acknowledge the reviewer’s valid concern regarding the strength of our claims concerning the practical implications of our findings. Recognizing that these implications warrant further empirical investigation, and in adherence to the scientific rigor of the present study, we have chosen to remove this claim from the current manuscript.

2 - The introduction references Zipf’s seminal works but fails to include more recent and critical discussions on the principle of least effort, optimal coding, information theory, two regimes in Zipf’s law and other relevant linguistic laws not studied here (Heaps-Herdan law, brevity law, Menzerath’s law...). In particular, some basic recent works are missed (see references), works that offer significant insights into the physical and mathematical origins of Zipf’s law that are highly relevant to the study (Debowski, 2020). These references should be included and discussed to establish a stronger theoretical foundation in this introduction, but not only this: other relevant linguistic laws should be analyzed in order to improve this work (i.e. see Torre et al., 2019).

**Authors:** We acknowledge the reviewer’s concern regarding the lack of recent theoretical discussions in the introduction. We have now expanded this section to include discussions of the principle of least effort, optimal coding, information theory, two-regime behavior in Zipf’s law, and other relevant linguistic laws (Heaps’ law, brevity law, Menzerath’s law). We have also incorporated the key reference of Debowski (2020) to address the physical and mathematical origins of Zipf’s law.

Specifically, we have referenced Torre et al. (2019) to provide a novel perspective on the potential physical origins of linguistic patterns, including the relationship between lognormality and speech production(also in literature review section). This reference adds depth to our understanding of the statistical regularities inherent in language and paves the way for a more nuanced analysis of word frequency distributions.

3 - While the introduction emphasizes the structured and formal nature of LGWRs, it does not sufficiently connect these features to the theoretical implications of Zipf’s law. The authors should discuss how document structure, specialized terminology, and political jargon might influence word frequency distributions. Incorporating this analysis, as well as the study of other linguistic laws like Zipf’s law of brevity, would deepen the reader’s understanding of how linguistic patterns in LGWRs align or deviate from Zipf’s law.

**Authors:** We have clarified in the introduction that the formal and structured nature of LGWRs, characterized by their use of specialized terminology and political jargon, may indeed influence word frequency distributions. We have discussed how these unique linguistic features could potentially align with or deviate from the predictions of Zipf’s law, emphasizing the importance of understanding these distributions within the context of formal political communication.

We acknowledge the reviewer’s suggestion to include other linguistic laws like Zipf’s law of brevity. However, we have chosen to maintain a focused approach on Zipf’s law as it is the central theme of our study. We believe that a detailed examination of Zipf’s law within the context of LGWRs provides a solid foundation for future research to explore the interplay between Zipf’s law and other linguistic regularities.

4 - The authors should also discuss the distinction between Zipf’s rank-frequency law and other related linguistic laws, that should be studied (see next points), like Zipf’s law of abbreviation: are the data studied following Zipf’s law of abbreviation? The two zipfian laws are sometimes conflated in the literature. A clearer delineation of these concepts would strengthen the theoretical framework. Specifically, please review if a two regime in Zipf’s law is found in the data (Ferrer i Cancho and Solé, 2001).

**Authors:** We have clarified the conceptual differences between Zipf’s rank-frequency law and Zipf’s law of abbreviation and other linguistic laws in the introduction and literature review sections. Specifically, we emphasized that while Zipf’s rank-frequency law describes the inverse relationship between word frequency and rank, Zipf’s law of abbreviation focuses on the inverse correlation between word frequency and word length. This distinction addresses potential conflation and provides a more nuanced theoretical framework for understanding linguistic regularities in LGWRs.

Given that LGWRs are composed of relatively short texts, employing the classic single double-logarithmic model was deemed the most appropriate analytical approach. Consequently, we did not divide the data into two regimes, as the brevity of the documents limits the practical applicability of such segmentation.

5-See point 2. A good literature review should include a general review on linguistic laws, and an extended review of Zipf’s law literature (see recent references in point 2). This is a key point of that review. For example, the works by Williams and cols. (2015) are discussed but not other related, comparing linguistic laws in speech and text (Piantadosi, 2014; Torre et al., 2019). Zipfian laws also include meaning-frequency laws, neither studied nor discussed here (see Català et al., 2024, and Català et al., 2021 and references therein).

**Authors:** We have significantly revised the Introduction(also in response to point 2) and Literature Review sections to address the concerns regarding the lack of theoretical foundation and the insufficient discussion of relevant literature. The revised Introduction and Literature Review sections have been expanded to include a wider range of pertinent works (Debowski, 2020; Font-Clos et al., 2013; Ferrer-i-Cancho et al., 2022; Kello et al., 2010; Semple et al., 2022; Ferrer i Cancho, 2007; Ferrer i Cancho & Solé, 2001; Piantadosi, 2014; Torre et al., 2019; Català et al., 2024; Català et al., 2021,etc.), and provides a more detailed and nuanced discussion of the Principle of Least Effort, optimal coding, information theory, two regimes in Zipf’s law, and other relevant linguistic laws such as Heaps’ law, Menzerath’s law, and the law of brevity. These laws, along with Zipf’s law, offer a framework for comprehending the statistical regularities inherent in language.

6-Authors claim that (lines 100-101): “Building on this research, the present study aims to further investigate the applicability of Zipf’s law within the context of Chinese local government work reports” but it is not clear what are the connection between the statistical work done and the suggestion of Chinese local government reports improvement. Please explain it better.

**Authors:**This study represents the first exploration of Chinese local government work reports as a political text through the lens of quantitative linguistics. We demonstrate that these reports adhere to Zipf’s Law and, further, analyze the deviation from the principle of least effort as reflected in their Zipf exponent. While our findings may contribute to linguistic refinements of such political documents, it is crucial to avoid overstating this contribution. The content and form of Chinese local government work reports are shaped by a multitude of factors, and improving the linguistic quality of these documents is likely a secondary consideration for local authorities.

7- The methodological section lacks detail on how the Jieba segmentation algorithm was fine-tuned for LGWRs, which likely include specialized political terminology. The authors should describe any preprocessing steps to ensure accurate segmentation and whether manual corrections were made.

**Authors:** We acknowledge the reviewer’s concern regarding the lack of detail on how the Jieba segmentation algorithm was fine-tuned for LGWRs, which include specialized political terminology. We have expanded the methodology section to describe our preprocessing steps, which included: (1) using regular expressions to remove punctuation, symbols, English characters, and numerals, and (2) constructing a custom Jieba dictionary with specialized terminology, political jargon, and relevant acronyms specific to LGWRs. Furthermore, the custom dictionary was created by manually reviewing representative documents and supplementing the original dictionary with specialized terms, optimizing segmentation accuracy.

8- In fact, it would be crucial to compare the analysis presented in this text with similar approaches applied to text on the same size (Corral et al., 2015) in other genres, such as literary works, scientific texts, or technical articles. This exercise could reveal common patterns, methodological differences, or unique perspectives that each type of content brings to the analysis of political texts. Furthermore, it would allow for an evaluation of how the tools or theoretical frameworks used in this work could be adapted or complemented in other discursive contexts, thereby fostering a broader and more multidisciplinary perspective. Could you explore these connections and share your insights?

**Authors:** We acknowledge the reviewer’s suggestion to compare our analysis with similar approaches applied to texts of comparable sizes and from different genres. While a comprehensive comparative study across different genres is beyond the scope of this current project, future research will explore these connections. We have added a statement in the conclusion section that further study of these types of comparative analyses could be valuable to strengthen the generalizability of our findings. Also the the conclusion section we have claimed that the methods and theoretical frameworks employed--natural language processing, double-logarithmic regression, and a custom Jieba dictionary--can be adapted and complemented for use in analyzing other discursive contexts, especially the Chinese text.

9- The choice of regression models could be appropriate but it is not adequately justified. The authors should explain why Stata’s regression methods were preferred over alternative statistical tools or machine learning approaches that might capture nonlinearities in the data, as well as other approaches (See Zipf’s section in Torre et al., 2019, and the supplementary data code). Why is not studied a double regime? (Ferrer I Cancho and Solé, 2001)?

**Authors:** We selected Stata’s regression tools because they are well-suited for estimating linear relationships in log-log transformed data, as is standard in studies of Zipfian distributions. The double-logarithmic regression approach provides a robust framework for examining the rank-frequency relationship and captures nonlinearities, particularly in the context of short texts like LGWRs. To enhance clarity, we have revised the methods section to explicitly address this choice and its alignment with similar studies, such as Torre et al. (2019).

As noted in the manuscript, LGWRs are relatively short texts. Given the limited word frequency ranges, splitting the data into two regimes would risk introducing artifacts or overfitting. This methodological consideration aligns with findings in Ferrer i Cancho and Solé (2001), where a two-regime analysis is more appropriate for larger or more heterogeneous datasets. To address this, we have expanded the discussion section to provide a detailed explanation of why a single-regime model was deemed most suitable for our corpus.

10- The results section provides a broad overview but lacks depth in exploring the observed heterogeneity in Pareto coefficients. Table 1 should be better explained. The authors should analyze whether these variations correlate with specific historical, regional, or policy-driven factors. For instance, do certain political or economic shifts influence the linguistic patterns in LGWRs? This should be improved studying other text sources (literary, scientific…) of the same size and comparing with the texts studied.

**Authors:** We have enhanced the discussion of the variations in Pareto coefficients presented in Table 2. Specifically, we analyzed potential correlations between these variations and key historical or policy-driven events, such as changes in leadership, the 18th National Congress of the Communist Party of China. These contextual factors have been linked to observable changes in linguistic patterns, such as shifts in the frequency of certain thematic vocabulary.

We have revised the description of Table 2 to provide a clearer explanation of its contents, emphasizing the significance of the range, mean, and standard deviation of Pareto coefficients across different provinces and time periods. This addition highlights how these metrics relate to linguistic variations and their underlying causes.

While the scope of this study is limited to LGWRs, we have suggested future research directions that involve comparing these results with texts from other genres of similar length (e.g., literary works, scientific texts, or technical articles). Such comparisons could reveal whether the observed linguistic patterns are unique to LGWRs or reflect broader trends in text production.

11-The authors note that LGWRs conform to Zipf’s law but do not critically examine the deviations and possible regimes (Ferrer i Cancho and Solé, 2001). Are these deviations systematic, and if so, what might they reveal about the dynamics of political communication? Are the meanings of the words related with frequency? With an analysis of the data using a Chinese dictionary (to obtain the number of meanings), authors can test the Zipf’s meaning-frequency law, to see if governement texts are more easy to understand that other kind of corpus (comparing oral corpus and written corpus, see Català et al., 2021).

**Authors:** We have revised the manuscript to include a more critical analysis of the observed deviations from Zipf’s rank-frequency law. Specifically, we investigated whether these deviations are systematic and what they may reveal about the dynamics of political communication in LGWRs.

We acknowledge the importance of examining the link between word frequency and semantic richness. While a full analysis of Zipf’s meaning-frequency law, requiring a detailed semantic analysis using a Chinese dictionary to determine the number of meanings for each word, was beyond the scope of the present study, we have included this as a critical avenue for future research in the Conclusion section. This explicit discussion of this significant limitation, and the potential implications of its future research, strengthens the study.

12-The discussion overgeneralizes the implications of the study, claiming that adherence to Zipf’s law can enhance report clarity and transparency. While this is an interesting hypothesis, it requires empirical validation. The authors should either provide evidence supporting their claims or frame it as a potential avenue for future research, comparing with other types of data.

**Authors:** We agree with the reviewers that this is an interesting hypothesis, but it needs to be further investigated by us in the future. However, in order to maintain the scientific rigor of this paper, we have removed this claim from the current manuscript.

13-The discussion could be enriched by addressing alternative explanations for the findings. For instance, how might the hierarchical structure of Chinese governance or regional linguistic variations influence the observed patterns?

**Authors:** We have explicitly addressed the heterogeneity across regions by analyzing the variations in Pareto coefficients in relation to historical, regional, and political factors, including the impact of the 18th National Congress of the Communist Party of China. The discussion now provides more nuanced interpretations of these regional differences and their potential causes.

14- The authors should consider whether their findings align with general principles in quantitative linguistics (Debowsky, 2020; Baixeries et al., 2013), and how this principle interacts with the specific context of political discourse.

**Authors:** We have expanded the discussion to situate our findings within the broader framework of quantitative linguistics. Specifically, We have expanded the discussion to explicitly link the observed β values in LGWRs to the balance between communicative efficiency and communication cost, as established in the broader literature on quantitative linguistics. We reference Debowsky (2020) and Baixeries et al. (2013) to support the argument that Zipfian distributions are not only universal but also reflective of the inherent efficiency and structure of human language systems.

15-The conclusion is clear but overly simplistic. The authors should briefly reiterate the study’s contributions to both quantitative linguistics and political communication while acknowledging its limitations. Future research directions, such as comparative studies with other countries’ government reports or different kinds of texts/corpora, should be suggested.

**Authors:** We acknowledge your comment that the conclusion was overly simplistic and have revised it accordingly. In the revised manuscript, we have specifically reiterated contributions, acknowledged limitations, and suggested future directions. We believe these changes have significantly improved the conclusion, making it more nuanced and informative while remaining concise.

16-Final comment. The authors can be pioneers in this type of analysis of linguistic laws in political discourse, which is why I invite a deep revision of this article and its subsequent re-submission. Congratulations and thank you for studying linguistic laws in this interdisciplinary way.

**Authors:** We are grateful for the reviewer’s positive feedback and encouragement. We hope that the changes made in this re-submission, along with the detailed explanation conveyed in the rebuttal letter, are sufficient to propose publication. Thank you once again for the valuable feedback.
