# Supplementary material for: Zipf’s law in China’s local government work reports: A 21-year study using natural language processing and regression analysis
Source: PLoS One. 2025 May 20;20(5):e0324713. doi: 10.1371/journal.pone.0324713 (PMC12091883; doi:10.1371/journal.pone.0324713)
Supplement: Attachment — Submitted filename: PONE-D-24-44352_review report 2025 0113 BL.doc [file pone.0324713.s003.doc]

# PLOS ONE

**Manuscript Review Report**

Manuscript #: PONE-D-24-44352

Title: Zipf’s Law in China’s Local Government Work Reports: A 21-year Study Using Natural Language Processing and Regression Analysis

Reviewer # BL

Report Date: 2025 01 13

This paper aims to test whether the content of government work reports in China adheres to Zipf’s law. Although the data set appears to be impressively collected from 31 provinces and areas over 21 years in span, the study should motivate the study in the Introduction section by clearly explaining why the content of government reports (in China or any other country) should (or should NOT) adhere to Zipf’s law, and what significance the study has to offer, rather than just testing the Zipf’s law in a specific context. The last sentence on Page 3 in lines 55-59 (termed as P3.55-59) is NOT the contribution of the study per se, but rather a repetition of the database and the aim of testing if the content of government work reports in China adheres to Zipt’s law as stated above.

Other more specific comments along the text are listed below:

1. P3.64-66: “…word frequency distribution in several Indian languages, including Hindi, Marathi, and Kannada, adheres to Zipf’s rule, demonstrating its universal applicability” is way too strong a conclusion. A more appropriate conclusion, at most, could be “demonstrating its POTENTIAL universal applicability in Indian languages”. To claim so, you need to make a case that these Hindi, Marathi, and Kannada languages are representative among N (you need to specify this number also) Indian languages, whereas N must NOT be too many larger than the "three" mentioned above. Otherwise, how can one justify using three out of, say, 10 (I suspect N is not a tiny number) Indian languages to conclude the result based on three languages could demonstrate its universal applicability for the many more different languages in India? Similarly, you need to make an even more complex case to conclude that Zipf’s rule is universally applicable to all the known languages in the world.

2. P4.74-75: Do you mean Tibetan is one kind of Chinese languages? If so, please define what you mean by the Chinese language in your study because Tibetan is an entirely different language than the popular Mandarin Chinese in written and dialogue. Do you mean the language(s) used in China (People's Republic of China)? If so, then, in P6.117-118 including Taiwan on the list is inaccurate (at least controversial). For non-political academic papers like this, it is better to avoid such a statement.
3. P7.141 & P8.165: Please clarify the potential inconsistency of the definitions of the Pareto Coefficient in these two places. P7.141 states, “with lnC representing the Pareto coefficient. The theoretical value of β remains 1” as in the equation on P7.143, whereas P8.165 states “The calculated Pareto coefficients (β values)…”.  If lnC is the Pareto Coefficient, please explain why the calculated Pareto coefficients are the β values. In P8.165, are you referring to the estimated values of the Regression coefficients? In either case, as reported in Table 1 (Pp.10-12.216-217), the maximum values of Pareto coefficients estimated by the regressions are all significantly below 1, please explain how these results are used to support the conclusion that “Chinese local government work reports generally 230 conform to Zipf’s law” (P13.230).
4. Please elaborate on the study's theoretical and practical contributions. You can use some of the materials reported in the Conclusion section. Besides, you can emphasize the implications of the different findings among different provinces/regions beyond an overall conformity to Zipf's law.

I hope these are useful for you to refine the paper.
