# Supplementary material for: Zipf’s law in China’s local government work reports: A 21-year study using natural language processing and regression analysis
Source: PLoS One. 2025 May 20;20(5):e0324713. doi: 10.1371/journal.pone.0324713 (PMC12091883; doi:10.1371/journal.pone.0324713)
Supplement: Attachment — Submitted filename: Response_to_Reviewers_auresp_2.doc [file pone.0324713.s007.doc]

Dear Editor and Reviewers,

Thank you for your insightful comments and suggestions on our manuscript, “Zipf’s Law in China’s Local Government Work Reports: A 21-Year Study Using Natural Language Processing and Regression Analysis”. We really appreciate your time and effort dedicated to evaluating the work and find the feedback invaluable in strengthening the manuscript. We have carefully addressed each point raised and believe the revisions significantly enhance the study’s rigor, clarity, and overall impact. All changes have been highlighted in red in the manuscript in response to the editor or reviewers’ comments. Below, you will find our detailed explanations and responses to each of your suggestions. The editor or reviewers’ comments are marked in blue, while our replies are presented in black.

**Analysis Suggestions**

1. **Regional Differences Need Better Storytelling:**
   - As noted by reviewers, it is hard to discern regional differences from the current presentation. To address this:
     - Plot the **trend of the top 20%** (e.g., provinces/mandates with the highest Zipf coefficients) and **contrast it with the bottom 20%.**
     - Perhaps the differences are between rural and urban regions? (As an example of a paper that tells a visual story nicely in PLOS ONE: Shankar, S.G., Miller, J.M., and Balakrishnan, P.V. (2020), "Evolutionary disruption of S&P 500 trading concentration: An intriguing tale of a financial innovation," PLOS ONE, 15(3), p.e0230393, is a potentially useful template for describing and studying evolutionary changes in the power law coefficient.)
     - To this end, **improve Figure 2 representation**:
       - The current Figure 2 is a blur of dots and does not effectively convey meaningful insights. Consider an alternative representation that highlights trends or patterns more clearly.
     - Use such comparisons to tell a more compelling story. For example:
       - Are provinces/mandates evolving in lockstep?
       - Are Zipf coefficients for documents from different regions evolving differently?
       - Or are all changes seemingly random?

**Authors:** Specifically, we have carefully considered the editor/reviewer’s comments regarding the presentation of regional differences and have made significant revisions. To address the specific request of comparing the “top 20%” to the “bottom 20%” of Pareto coefficients and to complement the original data presentation, we have added two new figures (Figures 4 and 5). These new figures provide a more effective visualization of the distribution of Pareto coefficients from the highest and lowest ends of the dataset. While these figures initially appeared to allow for any direct conclusions related to region, a textual analysis reveals the complex interplay of temporal effects of province-specific factors that is of key interest to the paper.

**2.Comparison with Consequential Paper:**

How do these findings compare with the statement by Naranan & Balasubrahmanyan (1998)?

"A word frequency analysis of a text can reveal nothing about its characteristics (author, language, style, type of literature, etc.). The only exception appears to be Shakespeare with ζ = 1.6; this fact has indeed been exploited in attempts to resolve some controversies about authorship of certain texts (Thisted & Efron, 1987)."

Feedback: Discuss whether your findings align with or challenge this claim in your context, particularly regarding whether regional or stylistic differences are discernible in Zipf coefficients.

**Authors:** We thank the editor/reviewer for their comment and have revised the manuscript to show that our word frequency analysis can reveal stylistic/thematic differences in specific contexts (Chinese political reports), contradicting their claim. We acknowledge that government work reports, owing to their standardized format, are not readily attributable to individual authorship, a factor often associated with stylistic variation. We further concede that in Zipf’s law, β-value has a theoretical value of 1, but empirically it deviates, partly due to text length (e.g., Shakespeare’s longer texts, Chinese political repors’ shorter texts), though our data suggests text length is not the sole factor.

**Motivation and Contribution**

1.Clarify the Contribution of the Study:

Clearly articulate how this study advances knowledge beyond existing research on Zipf’s law in Chinese language and documents.

Revise the last sentence on Page 3, Lines 55-59 (P3.55-59), to reflect the actual contribution of the study instead of repeating the dataset and aim.

Differentiate this study by highlighting your novel aspects (e.g., unique dataset, focus on local government work reports, or insights into political communication).

Incorporate findings from regional trends and comparison with prior research to show unique insights into regional or stylistic differences in political communication and linguistic evolution.

**Authors:** Thank you for your insightful feedback on the motivation and contribution of our study. We acknowledge that the previous version of the manuscript could have more clearly articulated our novel contributions. We have revised the manuscript to address your concerns. The manuscript is now revised to underscore the study’s unique aspects: the previously unexplored dataset, its focus on formalized political discourse, and methodological refinements, including a custom dictionary. We also note that our work challenges existing claims regarding the lack of relationship between word frequency and document characteristics, specifically by showing correlations between variations in Pareto coefficients and regional and political factors. We show that this unique methodological approach establishes a new baseline for the study of formal political documents by using existing quantitative linguistic methodology in a new way. We also state that our analysis provides unique insights into political language, builds upon existing research, and provides new avenues for future investigation.

We have revised the final sentence on Page 3(now Page 4, Line 98-102) to more accurately reflect our study’s contribution, moving beyond simply restating the dataset and aim.

2. Elaborate on Contributions:

Emphasize how findings contribute to understanding regional linguistic styles or political communication, if you find any after your revised analysis.

Highlight implications of differences among provinces/regions beyond overall conformity to Zipf’s law.

**Authors:** We acknowledge the importance of highlighting how our findings contribute to understanding regional linguistic styles or political communication, and of emphasizing the implications of differences among provinces/regions beyond overall conformity to Zipf’s law. We have made significant revisions to the manuscript to more directly address these points, which is now well highlighted. Key additions include:

In the Introduction, we added a paragraph discussing how the structured nature of government reports might lead to deviations from Zipf’s law but also prioritize common vocabulary. We also mention that the analysis reveals regional distinctions in linguistic patterns. In the Results section, we added a part about the lack of regional grouping in Pareto coefficients, indicating province-specific factors are more influential. In the Conclusion, we reveal that broad regional classifications are insufficient to explain linguistic styles in Chinese government work reports, it highlights the significance of province-specific and temporal factors, opening avenues for more granular analysis of political communication.

**Specific Minor Comments**

**1.Typo in Line 220:**

Correct “approximately1.” to “approximately 1.” (space missing).

**Authors:** Corrected, with thanks.

**2.Delete Lines 223-224:**

Remove: "While machine learning methods excel at predicting future trends, this study focuses on historical data and the relationships between variables." Reason: Adds nothing to the overall context.

**Authors:** We agree and have removed the sentence.

**3.Claim on Universal Applicability:**

Reword “demonstrating its universal applicability” to “demonstrating its potential universal applicability in Indian languages” (P3.64-66).

**Authors:** We agree and have reworded the sentence.

**4.Definition of Chinese Language and Scope:**

Clarify whether Tibetan is treated as a “Chinese language” or a distinct language used in China (P4.74-75).

Define what “Chinese language” encompasses (e.g., Mandarin, regional dialects, languages spoken within the People's Republic of China).

**Authors:** we agree with the reviewer’s important points regarding the definition of “Chinese language” and the scope of our study in relation to the inclusion of Tibetan. We now explicitly define “Chinese language” in the manuscript as Standard Mandarin, the official language of the People’s Republic of China.

In the literature review, we have further clarified the status of Tibetan, explicitly stating that “Tibetan ( a distinct language used in China)” was cited as an example of a previous study on Zipf’s Law

5. IMPORTANT — Delete Politically Charged Aspects:

Delete mention of Taiwan (P6.117-118) to avoid controversial statements in a non-political academic paper. Best is to delete names of all provinces you have NOT studied. (see Reviewer #4 as well)

**Authors:** We understand the reviewer’s concerns regarding the inclusion of potentially politically charged aspects. We have therefore removed all mention of Taiwan and the names of Hong Kong and Macau, which are not included in the corpus from the manuscript.

6.Inconsistencies in Pareto Coefficient Definitions:

Clarify (see Reviewer #4) whether the Pareto coefficient is represented by lnC or β in the manuscript (P7.141 & P8.165).

Explain the relationship between the calculated β values in regressions and the theoretical value of the Pareto coefficient (β= 1).

Address why the maximum β values reported in Table 1 are significantly below 1 and reconcile this with the conclusion that Chinese local government work reports conform to Zipf’s law.

**Authors:** Thank you for your careful review and for highlighting the need for clarification regarding the Pareto coefficient. In the manuscript,βconsistently represents the Pareto coefficient, not the constant term “C”(InC is not mentioned in the manuscript). We clarify the relationship between the theoretical (β=1) and empirical values, noting that their results (averageβ≈0.819) show a general tendency toward Zipf’s Law, not perfect adherence. We also explain that maximumβvalues being below 1 indicate systematic deviations caused by content concentration, reflecting political, regional, and report-specific influences. we believe that the revised manuscript and explanations adequately address the reviewer’s concerns.

7.Decimal Places in Lines 260-261:

Reduce excessive decimal places in the coefficient range (e.g., “0.6678291 to 0.9252278”) to a more reasonable number (e.g., 2 or 3).

**Authors:** We agree and have reduced excessive decimal places to 3

8.Overclaim in Line 288:

Avoid overstating conclusions by revising: “indicating that while most reports conform to Zipf’s law.”

**Authors:** We agree and have revised the sentence.

9.Engage with Suggested Literature: Reference the suggested article (https://www.colips.org/journals/volume18/JCLC_2008_V18_N1_04.pdf) and clarify how this study complements, differs from, or builds upon it.

**Authors:** Thank you for the suggested literature. We have carefully reviewed the suggested article by Xiao (2008) and have made revisions to explicitly address its relevance to our study. Revisions now explicitly engage with Xiao (2008), citing it in the literature review and discussing how our study differs by using a unique dataset (political reports), a custom dictionary, and by analyzing temporal and regional variations. The revised manuscript makes clear that our study complements previous work by extending the findings to a different kind of text, and also builds upon previous findings by investigating the limitations of previous research.

**Reviewer #2**

Lines 95-97. Authors claim that “This investigation contributes to a deeper understanding of the applicability of Zipf’s law within the context of formalized political communication.” Please clarify shortly how, in what way quantitative linguistics can do that, to explain this to a broader audience.

**Authors:** We appreciate your comment regarding the need to clarify how quantitative linguistics contributes to a deeper understanding of Zipf’s law within the context of formalized political communication, and have revised the manuscript accordingly. In the revised manuscript, we have added a more detailed explanation in the Introduction and Conclusion sections outlining how quantitative linguistics contributes to understanding formalized political communication.

Line 116. Torre et al. ->Torre et al. (2019). Please review all the manuscript to add year of publication when quoting direct name of author’s paper.

**Authors:** corrected, with thanks.

Line 138. Authors claim “In summary, whereas understanding of Zipf’s law was initially simplistic,...” I think it is better "In summary, whereas Zipf’s law was mathematically simple,...” because maths proposed by Zipf are easy but not deep concepts, in my opinion.

**Authors:** corrected, with thanks.

- Line 141. Menzerat’s -> Menzerath’s

**Authors:** corrected, with thanks.

- Section 3.2. please clarify how the transition point between two zipfian regime is found.

**Authors:** We appreciate the reviewer’s insightful observation concerning the lack of a clearly defined transition point between Zipfian regimes in our analysis. Our analysis, as reported, did not reveal a clear transition point. We believe this is largely due to the relatively short length of the government work reports in our corpus, making the identification of a transition point difficult. While we acknowledge this limitation, future research could explore this further using a larger dataset or employing alternative methodologies to analyze low-frequency words.

Finally, I encourage the authors to continue studying other linguistic laws in the materials they have (as they suggest they will do in the future work), as well as to analyze the semantics of political communications, something very important socially.

**Authors:** We are grateful for the reviewer’s positive feedback and encouragement. We fully agree with the reviewer that exploring other linguistic laws and delving into the semantics of political communication are crucial for a more comprehensive understanding of these documents. Our current study provides a foundation for these future avenues of research, and we are eager to pursue these directions in our subsequent work.
