# Supplementary material for: Zipf’s law in China’s local government work reports: A 21-year study using natural language processing and regression analysis
Source: PLoS One. 2025 May 20;20(5):e0324713. doi: 10.1371/journal.pone.0324713 (PMC12091883; doi:10.1371/journal.pone.0324713)
Supplement: Attachment — Submitted filename: PLOSONE-ZIPF-CHINA-EDITOR-R2.docx [file pone.0324713.s006.docx]

PLOS ONE: ZIPF CHINA REVIEW-R2

ACDEMIC EDITOR COMMENTS

**Rank-Frequency Law in Word Distributions (Zipf’s Law):** The **Rank-Frequency Law**, also known as **Zipf’s Law**, describes the relationship between the **frequency** of a word in a corpus and its **rank** in a descending ordered list of word occurrences. The **Rank-Size Law** as you have corerctly stated in equation (on Line 226) states that the frequency ($S$) of a word is inversely proportional to its rank ($R$) raised to a power **α**:

$$S=CR^{-\alpha}$$

where: - $S$ = frequency of the word in the corpus - $R$ = rank of the word (e.g., the most frequent word in your corpus is ranked 1, the second-most frequent is ranked 2, etc.) - $C$ = a constant (scaling factor) - $\alpha$ = **Zipf coefficient** (slope of the rank-frequency distribution)

Taking the natural logarithm on both sides:

$$\log\left( S \right)=log\left( C \right)-\alpha\log\left( R \right)$$

or equivalently:

$$\log\left( R \right)=\frac{\log\left( C \right)}{\alpha}-\frac{1}{\alpha}\log\left( S \right)$$

This is the equivalent of the equation you have used for your MLE or **OLS regression estimation** of **α**, where:

$$\log\left( R \right)=\beta_{0}+\beta_{1}\log\left( S \right)$$

with: $\beta_{0}=\frac{\log\left( C \right)}{\alpha}$ ; $\beta_{1}=-\frac{1}{\alpha}$

Thus, solving for **α**:

$$\alpha=-\frac{1}{\beta_{1}}$$

But it seems as the reviewer has pointed out, this reciprocal transformation may have not been implemented. In either case, your setup and writing of this aspect is rather confusing as you move from Zipf’s Law to Pareto Distribution etc.

I suggest that given the focus of your paper is on ZIPF’s LAW as you have used in the title of your manuscript, you stick with rporting and analyzing and plotting the Zipf Coefficients. For the connection between Zipf and Pareto please see the note by Lada A. Adamic. This tutorial explains the relationship between Zipf’s law, power-law distributions, and Pareto’s law, highlighting how they describe similar phenomena in different ways.

**Source:** <https://web.cs.dal.ca/~shepherd/courses/csci4141/zipf/ranking.html> **Zipf, Power-laws, and Pareto - a ranking tutorial, Lada A. Adamic.** This, a higher Zipf coefficient corresponds to a more uneven distribution with a lower Pareto coefficient; Conversely, a lower Zipf coefficient reflects a more even distribution with a higher Pareto coefficient.

**This leads to an easier interpretation of the Zipf Coefficient (α).**

- **If α ≈ 1**, the data follows **Zipf’s Law**, meaning word frequencies follow a typical rank-size rule.
- **If α > 1**, the distribution is **steeper**, meaning a few high-frequency words dominate the text.
- **If α < 1**, the distribution is **flatter**, meaning word frequencies are more evenly distributed.

I recommend that you rewrite this so we have a clean paper with respect to Zipf’s Law.

**Zipf Coefficients movements Need Better Storytelling:**

- As noted by reviewers, it is not iclear as to what your findings are. At this stage, I am willing to suggest that the major contribution hs been to conduct a challenging and exahustive analysis to it is hard to discern regional differences from the current presentation. To address this:
  - Plot the **trend of the top (10%) 20%** with the highest Zipf coefficients and **contrast it with the bottom (10%) 20%. I conducted this analysis (based on the limited data you have made available of the coefficeints in excel file).** I present the plot below. There is a significant divergence in the two plots. I am also able to see a natural break-point from this plot at about year 2011 or so. I used as template a paper in *PLOS ONE*: Shankar, S.G., Miller, J.M., and Balakrishnan, P.V. (2020), “Evolutionary disruption of S&P 500 trading concentration: An intriguing tale of a financial innovation,” *PLOS ONE,* 15(3), p.e0230393, which was very useful for describing and studying evolutionary changes in the Zipf coefficient.
  - Please do this carefully, but using the ZIPF Coefficients, so that the connection can be made easier to the Principle of Least Effort.
  - Perhaps the differences are between rural and urban regions? Please indicate in your table with one identifier as to which are Rural and which are Urban.
  - Unfortunately, all Figure 2 to 4 are nothing but a blur of blue dots and do not effectively convey meaningful insights. Consider an alternative representation that highlights trends or patterns more clearly. Please replace them with the suggested plots. Use color coding to make them tell a visual story. Use such comparisons to tell a more compelling story. For example:
    - Are provinces/mandates evolving in lockstep?

### [PLOS Data policy](http://www.plosone.org/static/policies.action#sharing)

A big hurdle is the the authors have NOT made all data underlying the findings in their manuscript fully available. The [PLOS Data policy](http://www.plosone.org/static/policies.action#sharing) requires authors to make all data underlying the findings described in their manuscript fully available without restriction, with rare exception I was able to access ONLY their reported estimate of the Pareto Coefficients. It was not possible for me to replicate or help them enhance their analysis. The only folder to a region is Beijing and that is empty.

**Please make sure to upload the corpus frequencies data.**

STYLISTIC AND SMALLER EDITS / COMMENTS:

Lines 229-230 are confusing the metrics and can be eliminated.

Line 240. Reference to “Torrre et al (2019) [13]” is strange, pick one citation reference style.

Line 337 - 340: DELETE “Our findings directly challenge the assertion by Naranan and Balasubrahmanyan (1998) that word frequency analysis reveals nothing about a text’s characteristics (e.g., author, language, style, and type of literature) [37].” Currently, the paper has not made a substantive contribution on these matters. At best you have some suggestive findings that need investigation.

STATA MLE ESTIMATION: Please clearly specify as to what package(s) were used to estimate the coefficients. Are you using “ml maximize”? How did you define the likelihood function? Sample code use for the MLE should be provided to ensure replication.

In Table 1 Descriptive statistics on the length of government work reports, please clarify the units. I assume that these are number of unique “words” in each corpus-year?

### **Other Recommendations for Enhancing the Analysis and Presentation**

**1. Strengthening the Visual Narrative**

the authors should **plot the top 20% and bottom 20% of Zipf values** across all regions for each year; **comparison of the 10% and 20% groupings** in a single plot would allow for better trend visualization. Overlaying both **10% and 20% plots** on the same figure will help in assessing the stability of rankings. In addition, I was able to identify the natural brak point which seems to correspond with the 18th National Congress of the Communist Party of China in 2012.

**2. Identifying Key Regions in Extreme Categories**

- The authors should **determine the specific regions that consistently appear in the top 10% (20%) and bottom 10%** (20%) of Zipf values over time. A **table** should be included to highlight persistent high- and low-ranking regions.

#### **3. Regional Movement and Stability Analysis**

- Additionally, it would be useful to **identify which regions frequently transition between these groups**. If possible see if there are some geographical or political aspects as to why this occurs. A **table summarizing how many times each region appeared in the top 20% or bottom 20% over the years** should be included. Another set should identify **regions that never appeared in either extreme**, as this suggests stability.
- **Persistence and Volatility Metrics:** The authors should **calculate persistence scores** to rank regions by how consistently they appear in either the top or bottom 20%. Conversely, the authors should identify **regions that frequently switch between top and bottom groups** to highlight volatility. The discussion should explore **why some regions are stable while others are more dynamic** in their rankings.
