# Supplementary material for: Zipf’s law in China’s local government work reports: A 21-year study using natural language processing and regression analysis
Source: PLoS One. 2025 May 20;20(5):e0324713. doi: 10.1371/journal.pone.0324713 (PMC12091883; doi:10.1371/journal.pone.0324713)
Supplement: Attachment — Submitted filename: response letter.docx [file pone.0324713.s008.docx]

Dear Editor and Reviewers,

We would like to express our sincere gratitude for your thoughtful comments and suggestions regarding our manuscript, “Zipf’s Law in China’s Local Government Work Reports: A 21-Year Study Using Natural Language Processing and Regression Analysis”. We truly appreciate the time and effort you have invested in reviewing our work, and your feedback has been extremely valuable in improving the manuscript.

We have carefully considered each of your points and made revisions that we believe greatly enhance the study’s rigor, clarity, and overall quality. The changes we have made are highlighted in red in the manuscript, in direct response to your feedback. In the following sections, we provide detailed explanations of how we have addressed your comments. The reviewer and editor’s original comments are marked in blue, with our responses in black.

**Rank-Frequency Law in Word Distributions (Zipf’s Law):** The **Rank-Frequency Law**, also known as **Zipf’s Law**, describes the relationship between the **frequency** of a word in a corpus and its **rank** in a descending ordered list of word occurrences. The **Rank-Size Law** as you have corerctly stated in equation (on Line 226) states that the frequency ($S$) of a word is inversely proportional to its rank ($R$) raised to a power **α**:

$$S=CR^{-\alpha}$$

where: - $S$ = frequency of the word in the corpus - $R$ = rank of the word (e.g., the most frequent word in your corpus is ranked 1, the second-most frequent is ranked 2, etc.) - $C$ = a constant (scaling factor) - $\alpha$ = **Zipf coefficient** (slope of the rank-frequency distribution)

Taking the natural logarithm on both sides:

$$\log\left( S \right)=log\left( C \right)-\alpha\log\left( R \right)$$

or equivalently:

$$\log\left( R \right)=\frac{\log\left( C \right)}{\alpha}-\frac{1}{\alpha}\log\left( S \right)$$

This is the equivalent of the equation you have used for your MLE or **OLS regression estimation** of **α**, where:

$$\log\left( R \right)=\beta_{0}+\beta_{1}\log\left( S \right)$$

with: $\beta_{0}=\frac{\log\left( C \right)}{\alpha}$ ; $\beta_{1}=-\frac{1}{\alpha}$

Thus, solving for **α**:

$$\alpha=-\frac{1}{\beta_{1}}$$

But it seems as the reviewer has pointed out, this reciprocal transformation may have not been implemented. In either case, your setup and writing of this aspect is rather confusing as you move from Zipf’s Law to Pareto Distribution etc.

I suggest that given the focus of your paper is on ZIPF’s LAW as you have used in the title of your manuscript, you stick with rporting and analyzing and plotting the Zipf Coefficients. For the connection between Zipf and Pareto please see the note by Lada A. Adamic. This tutorial explains the relationship between Zipf’s law, power-law distributions, and Pareto’s law, highlighting how they describe similar phenomena in different ways.

**Source:** <https://web.cs.dal.ca/~shepherd/courses/csci4141/zipf/ranking.html> **Zipf, Power-laws, and Pareto - a ranking tutorial, Lada A. Adamic.** This, a higher Zipf coefficient corresponds to a more uneven distribution with a lower Pareto coefficient; Conversely, a lower Zipf coefficient reflects a more even distribution with a higher Pareto coefficient.

**This leads to an easier interpretation of the Zipf Coefficient (α).**

- **If α ≈ 1**, the data follows **Zipf’s Law**, meaning word frequencies follow a typical rank-size rule.
- **If α > 1**, the distribution is **steeper**, meaning a few high-frequency words dominate the text.
- **If α < 1**, the distribution is **flatter**, meaning word frequencies are more evenly distributed.

I recommend that you rewrite this so we have a clean paper with respect to Zipf’s Law.

**Authors:** We acknowledge your point about the potential confusion between the discussion of Zipf’s Law and the Pareto distribution. Indeed, the reciprocal transformation, as outlined in the editor’s comment, was not clearly implemented or explained in our original presentation. This has likely caused some confusion regarding the connection between Zipf’s Law and the Pareto distribution in the context of our regression analysis. We appreciate your suggestion to focus specifically on Zipf’s Law in the paper, as it will allow for a more coherent and streamlined analysis.

To address this issue, we have revised the manuscript to ensure that the discussion remains centered on Zipf’s Law. We have removed references to the Pareto distribution where unnecessary, and have reframed the analysis to focus on the interpretation and application of the Zipf coefficient. In particular, we now emphasize the relationship between the Zipf coefficient and the distribution of word frequencies within the government work reports, as follows:

1. **Interpretation of the Zipf Coefficient:** We highlight that if the Zipf coefficient (β) is approximately 1, the distribution follows Zipf’s Law, with a typical rank-size rule. If β > 1, the distribution becomes steeper, indicating dominance by a few high-frequency words. Conversely, if β < 1, the distribution is flatter, signifying a more even distribution of word frequencies across the corpus.
2. **Simplification of the Regression Model:** We have revised our explanation of the regression models used in our analysis to focus solely on the calculation and significance of the Zipf coefficient, eliminating any unnecessary complexity related to the Pareto distribution. We have also clarified the process of estimating β through the double-logarithmic regression model, ensuring a more straightforward interpretation of the Zipfian distributions observed in our corpus.

Zipf Coefficients movements Need Better Storytelling:

As noted by reviewers, it is not clear as to what your findings are. At this stage, I am willing to suggest that the major contribution has been to conduct a challenging and exhaustive analysis to it is hard to discern regional differences from the current presentation. To address this:

Plot the trend of the top (10%) 20% with the highest Zipf coefficients and contrast it with the bottom (10%) 20%. I conducted this analysis (based on the limited data you have made available of the coefficeints in excel file). I present the plot below. There is a significant divergence in the two plots. I am also able to see a natural break-point from this plot at about year 2011 or so. I used as template a paper in PLOS ONE: Shankar, S.G., Miller, J.M., and Balakrishnan, P.V. (2020), “Evolutionary disruption of S&P 500 trading concentration: An intriguing tale of a financial innovation,” PLOS ONE, 15(3), p.e0230393, which was very useful for describing and studying evolutionary changes in the Zipf coefficient.

Please do this carefully, but using the ZIPF Coefficients, so that the connection can be made easier to the Principle of Least Effort.

Perhaps the differences are between rural and urban regions? Please indicate in your table with one identifier as to which are Rural and which are Urban.

Unfortunately, all Figure 2 to 4 are nothing but a blur of blue dots and do not effectively convey meaningful insights. Consider an alternative representation that highlights trends or patterns more clearly. Please replace them with the suggested plots. Use color coding to make them tell a visual story. Use such comparisons to tell a more compelling story.

**Authors:** We appreciate your recognition of the challenges involved in conducting this exhaustive analysis. We also understand the need for clearer storytelling to effectively convey the findings, particularly regarding the regional differences in the Zipf coefficients. In light of your comments, we have thoroughly reviewed and revised the manuscript to address the points raised. Below, we detail the specific modifications made in response to your feedback.

**1. Clarifying Findings and Improving Visual Representation:** We acknowledge that the original presentation of our findings, particularly in Figs 2 to 5, was not as effective as it could have been in conveying meaningful insights. In response to your comments, we have made substantial revisions to the figures and analysis to better highlight trends and patterns. Specifically:

Revised Fig 2: We have modified this figure to show the evolution of the Zipf coefficients for each provincial-level region over time. This allows for a clearer visualization of regional trends and variations.

Revised Fig 3: We have replaced the original figure with a combination of histograms and kernel density estimation of the Zipf coefficients. This provides a more intuitive understanding of the distribution and changes in the coefficients over time.

Revised Fig 4: This figure now presents the average Zipf coefficients for each year. The plot clearly shows an upward trend after 2011, confirming this year as a critical turning point in the data.

Revised Fig 5: We have added a figure showing the distribution of Zipf coefficients across quintiles. This analysis further supports the identification of 2011 as a key inflection point.

**2. Additional Analysis--Regional and Municipal Comparisons:** To further enrich our analysis and address potential regional differences, we have added two new figures:

Fig 6: This figure compares the Zipf coefficients across four major regions of China: Eastern, Central, Western, and Northeastern regions. The results show that the trends are largely similar across these regions, with no significant differences observed.

Fig 7: This figure compares the Zipf coefficients between centrally-governed municipalities (Beijing, Tianjin, Shanghai, and Chongqing) and non-municipal provincial-level regions. While the overall trends are similar, we found that the centrally-governed municipalities generally had higher Zipf coefficients during the periods 2003-2015 and 2020-2023, with only five years where their coefficients were lower than those of non-municipal provincial-level regions.

**3. Addressing Rural vs. Urban Distinction:** We appreciate your suggestion to explore potential differences between rural and urban regions. However, as the data used in our study is derived from annual government work reports, which are prepared at the provincial or municipal level, there is no direct distinction between rural and urban areas in the dataset. Therefore, we were unable to perform this specific analysis. We hope this explanation clarifies the limitation in addressing this particular point.

**4. Connection to the Principle of Least Effort：**We appreciate your guidance on connecting our findings to the Principle of Least Effort. As you noted, the Zipf’s Law represents the Principle of Least Effort, where a β value of 1 indicates perfect adherence to this principle. In our empirical analysis, we found that the β values for Chinese provincial government work reports are close to 1 but not equal to 1. This suggests that while the language used in these reports approaches the Principle of Least Effort, there is still room for improvement, as the system is not perfectly optimized.

### [PLOS Data policy](http://www.plosone.org/static/policies.action#sharing)

A big hurdle is the the authors have NOT made all data underlying the findings in their manuscript fully available. The [PLOS Data policy](http://www.plosone.org/static/policies.action#sharing) requires authors to make all data underlying the findings described in their manuscript fully available without restriction, with rare exception I was able to access ONLY their reported estimate of the Pareto Coefficients. It was not possible for me to replicate or help them enhance their analysis. The only folder to a region is Beijing and that is empty.

**Authors:** We sincerely apologize for the inconvenience caused by the incomplete data upload in the initial submission. Due to an error during the data upload process, the full dataset underlying the findings in our manuscript was not made available as intended. We deeply regret this oversight and appreciate the reviewer’s patience and understanding. In response to your comment, we have now carefully reviewed and re-uploaded the complete dataset, including all the raw data and processed files necessary to replicate our analysis.

STYLISTIC AND SMALLER EDITS / COMMENTS:

Lines 229-230 are confusing the metrics and can be eliminated.

**Authors:** Corrected, with thanks.

Line 240. Reference to “Torrre et al (2019) [13]” is strange, pick one citation reference style.

**Authors:** Corrected, with thanks.

Line 337 - 340: DELETE “Our findings directly challenge the assertion by Naranan and Balasubrahmanyan (1998) that word frequency analysis reveals nothing about a text’s characteristics (e.g., author, language, style, and type of literature) [37].” Currently, the paper has not made a substantive contribution on these matters. At best you have some suggestive findings that need investigation.

**Authors:** Corrected, with thanks.

STATA MLE ESTIMATION: Please clearly specify as to what package(s) were used to estimate the coefficients. Are you using “ml maximize”? How did you define the likelihood function? Sample code use for the MLE should be provided to ensure replication.

**Authors:** We sincerely thank the reviewer for their careful reading and valuable feedback regarding the estimation method used in our analysis. We would like to clarify that our regression analysis was conducted using the regress command in STATA, which employs the Ordinary Least Squares (OLS) method, not Maximum Likelihood Estimation (MLE). We apologize for any confusion caused by the initial description and have revised the manuscript to explicitly state this.

Code for Replication：reg log(S) log(R)

In Table 1 Descriptive statistics on the length of government work reports, please clarify the units. I assume that these are number of unique “words” in each corpus-year?

**Authors:** We sincerely thank the reviewer for their careful reading and valuable feedback regarding Table 1. We apologize for any confusion caused by the lack of clarity in the units used in the table. In response to the editor’s comment, we would like to clarify the following:

**Units in Table 1:** The values in Table 1 represent the total word count (i.e., the number of words, including repetitions) in each government work report for a given year, not the number of unique words. These word counts were calculated after preprocessing the text, including word segmentation using the Jieba library and removal of punctuation, special symbols, and non-Chinese characters.

**Clarification in the Manuscript:**We have revised the caption of Table 1 to explicitly state the units. The updated caption now reads: “Table 1. Descriptive statistics on the length (total word count) of government work reports.” Additionally, we have added a note below the table to further clarify:“Note: The length of each report is measured as the total word count, including repeated words, after preprocessing the text.”

Other Recommendations for Enhancing the Analysis and Presentation

1. Strengthening the Visual Narrative

the authors should plot the top 20% and bottom 20% of Zipf values across all regions for each year; comparison of the 10% and 20% groupings in a single plot would allow for better trend visualization. Overlaying both 10% and 20% plots on the same figure will help in assessing the stability of rankings. In addition, I was able to identify the natural brak point which seems to correspond with the 18th National Congress of the Communist Party of China in 2012.

**Authors:** We sincerely thank the reviewer for their valuable suggestion to enhance the visual narrative by plotting the top 20% and bottom 20% of Zipf values across all regions for each year, as well as comparing the 10% and 20% groupings in a single plot. We agree that such visualizations can provide deeper insights into trends and ranking stability. In our revised manuscript, we have addressed this suggestion by adding Fig 5, which illustrates the distribution of Zipf coefficients across quintiles (20% groupings) over time. This figure confirms 2011 as a critical inflection point, aligning with the 18th National Congress of the Communist Party of China in 2012, as noted by the editor. However, we did not overlay the 10% and 20% groupings in a single plot for the following reasons:

**1 Representativeness of 20% Groupings:** Our analysis revealed that the trends for the top and bottom 20% were already highly representative of the overall patterns. The differences between the top 10% and 20% (or bottom 10% and 20%) were not substantial enough to warrant separate visualization.

**2 Clarity and Conciseness:** Including both 10% and 20% groupings in a single plot could make the figure overly complex and less interpretable. Instead, we focused on presenting the quintile analysis in Fig 5, which provides a clear and concise representation of the trends.

We believe that Fig 5 effectively addresses the editor’s concern about trend visualization and stability. Additionally, the inflection point in 2011 is clearly highlighted, supporting the editor’s observation of its correspondence with the 18th National Congress of the Communist Party of China in 2012.

2. Identifying Key Regions in Extreme Categories

The authors should determine the specific regions that consistently appear in the top 10% (20%) and bottom 10% (20%) of Zipf values over time. A table should be included to highlight persistent high- and low-ranking regions.

**Authors:** while we appreciate this valuable suggestion, our revised manuscript does not include such a table for the following reasons:

1. **Lack of Distinct Regional Patterns:** Our analysis revealed that no clear regional patterns or characteristics distinguish the highest and lowest Zipf coefficients over time. For example, regions with the highest or lowest coefficients did not exhibit consistent geographical, economic, or political traits. This finding is further supported by Fig 6 in our revised manuscript, which shows that the trends in Zipf coefficients across China’s four major regions (Eastern, Central, Western, and Northeastern) are highly similar, with no significant regional differences.
2. **Limited Interpretability:** Given the lack of distinct patterns among regions in the extreme categories, a table highlighting persistent high- and low-ranking regions would not provide meaningful insights into regional variations. Instead, we focused on analyzing the temporal trends and inflection points, which revealed more significant and interpretable patterns. For instance, Fig 5 illustrates the distribution of Zipf coefficients across quintiles, confirming 2011 as a critical inflection point, while Fig 7 compares Zipf coefficients between centrally-governed municipalities and other provincial-level regions, revealing higher coefficients in municipalities during specific periods.

3. Regional Movement and Stability Analysis

Additionally, it would be useful to identify which regions frequently transition between these groups. If possible see if there are some geographical or political aspects as to why this occurs. A table summarizing how many times each region appeared in the top 20% or bottom 20% over the years should be included. Another set should identify regions that never appeared in either extreme, as this suggests stability.

Persistence and Volatility Metrics: The authors should calculate persistence scores to rank regions by how consistently they appear in either the top or bottom 20%. Conversely, the authors should identify regions that frequently switch between top and bottom groups to highlight volatility. The discussion should explore why some regions are stable while others are more dynamic in their rankings.

**Authors:** We thank the reviewer for their suggestion to analyze regional movement and stability. However, our revised manuscript does not include this analysis for the following reasons:

**1 National Policy Influence Over Regional Differences:** Our findings indicate that the linguistic patterns in Chinese local government work reports are heavily influenced by national policy directives rather than regional characteristics. The upward trend in Zipf coefficients after 2011, coinciding with the 18th National Congress of the Communist Party of China, reflects a nationwide shift toward more standardized and centralized policy communication. This trend is observed across all regions, further reducing the significance of regional differences.

**2 Focus on Temporal Trends and Overall Adherence to Zipf’s Law:** The primary focus of our study is to examine the overall adherence to Zipf’s law in Chinese local government work reports and to analyze temporal trends in Zipf coefficients over a 21-year period. Our results demonstrate a clear upward trend in Zipf coefficients after 2011, driven by national policy shifts rather than regional dynamics. Given this focus, we prioritized analyzing temporal trends and inter-regional similarities rather than regional mobility and stability.

**Specific Responses to Reviewer Comments:**

**Reviewer # BL**

3.P7.141 & P8.165: Please clarify the potential inconsistency of the definitions of the Pareto Coefficient in these two places. P7.141 states, “with lnC representing the Pareto coefficient. The theoretical value of β remains 1” as in the equation on P7.143, whereas P8.165 states “The calculated Pareto coefficients (β values)…”.  If lnC is the Pareto Coefficient, please explain why the calculated Pareto coefficients are the β values. In P8.165, are you referring to the estimated values of the Regression coefficients? In either case, as reported in Table 1 (Pp.10-12.216-217), the maximum values of Pareto coefficients estimated by the regressions are all significantly below 1, please explain how these results are used to support the conclusion that “Chinese local government work reports generally 230 conform to Zipf’s law” (P13.230).

Although the author responded that “In the manuscript, β consistently represents the Pareto coefficient, not the constant term “C”(InC is not mentioned in the manuscript)”, I think the author meant “lnC is NO LONGER mentioned in the R2 manuscript” because the author changed the empirical model of Zipf’s law from that displayed in R1 manuscript* to R2 manuscript (P12.223- 227) that shows “Zipf’s law is satisfied when the absolute frequency (n) of a word and its rank order (r) adhere to the following power-law relationship:

n ∝1/ 𝑟^𝛽^

where β is the Pareto coefficient. A value of β = 1 indicates perfect adherence to Zipf’s law.”

* In R1 manuscript, it stated in P7.138-144 “… Zipf’s law is as follows:

Rank = C/Freqβ

where the word frequency (Freq) is multiplied by the ranking (Rank), and a constant term (C) is added, with lnC representing the Pareto coefficient. The theoretical value of β remains 1.

The above formula can be logarithmically transformed by taking the logarithm of both sides:

ln(Rank)=lnC- βln(Freq)

This represents the empirical model of Zipf’s rule…”, whereas P8.165 states “The calculated Pareto coefficients (β values)…”

However, the author further states in the R2 manuscript (P12.228-P13.235) that

“Alternatively, Zipf’s law can be expressed as:

f(𝑛) ∝1/𝑛^𝛼^

where f(n) is the probability mass function of rank n, with α = 1 +1/β

Perfect adherence to Zipf’s law occurs when α = 2. When β = 1, the law simplifies to:

n × r = C

where C is a constant. This means the product of a word’s rank and frequency is constant:

the most frequent word (r = 1) is twice as frequent as the second (r =2), the third is three times less frequent than the first, and so on”.

Here appears a few critical questions:

3a. What does “f(n) is the probability mass function of rank n” mean?

3b. Does “n” represent Frequency or Rank in this equation? Please note that rank has been represented by “r” in other parts of the manuscript. Such kind of potential inconsistency should not exist.

3c. How does f(n) ∝1/𝑛^𝛼^ simplify to n × r = C, when β = 1?

3d. Is the regression coefficient that is reported the inverse of the Power Law coefficient? Why, or why not?

**Authors:** We sincerely thank the reviewer for their careful reading and valuable feedback regarding the definitions and consistency of the Pareto coefficient in our manuscript. We have revised the relevant sections to address these concerns.

The reviewer correctly identified inconsistencies in our terminology between the original and revised manuscripts. We thank the reviewer for bringing this to our attention and have thoroughly revised our mathematical notation to ensure consistency.

In our revised manuscript, we have standardized our terminology and notation. We now consistently use β to represent the Zipf coefficient. This coefficient is the critical parameter that determines how closely the word frequency distribution adheres to Zipf’s law.

We have eliminated the confusing reference to “lnC” that appeared in the original manuscript. The constant term c in our equations is simply a scaling constant.

Response to the 3 critical questions:

3a. Meaning of “f(n) is the probability mass function of rank n”

We acknowledge the confusion in our terminology. This was imprecise and has been corrected in the manuscript. The revised formulation now clearly states:

“Zipf’s law is satisfied when the absolute frequency (S) of a word and its rank order (R) adhere to the following power-law relationship:

S ∝ 1/R^β

where β is the Zipf coefficient.”

3b. Inconsistency in notation for frequency and rank

The reviewer correctly identified inconsistencies in our notation. In the revised manuscript, we have standardized our notation throughout:

S consistently represents the absolute frequency of a word

R consistently represents the rank order

β consistently represents the Zipf coefficient

We have thoroughly reviewed the entire manuscript to ensure this consistency is maintained.

3c. Simplification of the power law when β = 1

We have clarified the derivation in the revised manuscript. When β = 1, the equation:

$S\propto\frac{1}{R^{\beta}}$ becomes $S\propto\frac{1}{R}$

This can be rewritten as S × R = c (where c is a constant).

The revised manuscript now includes this derivation more explicitly to avoid confusion.

3d. Relationship between regression coefficient and power law coefficient

We appreciate the reviewer’s question about the regression coefficient. In our double-logarithmic regression model:

$$\log S_{it}=log c-\log R_{it}+\varepsilon$$

The estimated regression coefficient is indeed negative β. In the log-log regression, this appears with a negative sign because of the inverse relationship between frequency and rank. We have clarified in the manuscript that “the regression coefficient is negative β” to eliminate any confusion.

Regarding Values Below 1 and Conformity to Zipf’s Law

The reviewer correctly notes that our estimated Zipf coefficients are below 1 (average of 0.819). We have refined our conclusion to acknowledge this deviation from the theoretical value while emphasizing that this is consistent with empirical findings in other linguistic studies.

In the literature on Zipf’s law, perfect adherence (β=1) is rarely observed in real-world data. Our values are within the range commonly found in empirical linguistic studies, which supports our conclusion that “Chinese local government work reports generally conform to Zipf’s law, though with systematic deviations as commonly observed in empirical linguistic studies.”

We have references to support this interpretation, noting that Piantadosi (2014) and other researchers have found similar deviations while still considering the texts to follow Zipfian distributions.

We appreciate the reviewer’s detailed attention to our mathematical formulation, which has helped us to improve the clarity and consistency of our manuscript. We believe these revisions address all concerns raised and strengthen the overall quality of our work.
