# Supplementary material for: Zipf’s law in China’s local government work reports: A 21-year study using natural language processing and regression analysis
Source: PLoS One. 2025 May 20;20(5):e0324713. doi: 10.1371/journal.pone.0324713 (PMC12091883; doi:10.1371/journal.pone.0324713)
Supplement: Attachment — Submitted filename: response_letter_auresp_4.docx [file pone.0324713.s009.docx]

Dear Editor and reviewers,

Thank you very much for your encouraging feedback and for overseeing the review process of our manuscript. We sincerely appreciate your and the reviewers’ thoughtful comments and suggestions, which have helped us significantly improve the quality and clarity of the paper.

In the following sections, we provide detailed explanations of how we have addressed your comments. The editor or reviewers’ original comments are marked in blue, with our responses in black.

Journal Requirements:

Please review your reference list to ensure that it is complete and correct. If you have cited papers that have been retracted, please include the rationale for doing so in the manuscript text, or remove these references and replace them with relevant current references. Any changes to the reference list should be mentioned in the rebuttal letter that accompanies your revised manuscript. If you need to cite a retracted article, indicate the article’s retracted status in the References list and also include a citation and full reference for the retraction notice.

**Authors:** We have thoroughly checked the reference list and made the following modifications in response to the journal requirements:

1 We have removed reference [37] (Naranan & Balasubrahmanyan, 1998) because: The text passage citing this reference (“contradicting Naranan and Balasubrahmanyan's claim [37]”) was deleted following editor’s suggestions. The removed content was the only citation of this reference in our manuscript.

2 We have added DOI links and accessibility information for the following Chinese literature to improve reference completeness and accessibility:

Qin K. The applicability of Zipf’s law in ancient Chinese texts—taking bibliometrics of Dream Brook Sketchbook as an example. Shanxi Library J. 2020;42(4):52-59. doi:10.3969/j.issn.1004-1680.2020.04.009

Xiao H. On the applicability of Zipf’s Law in Chinese word frequency distribution. J Chin Lang Comput. 2008;18(1):33-46. Available from: <https://www.colips.org/journals/volume18/JCLC_2008_V18_N1_04.pdf>

We appreciate your guidance in improving the quality and reliability of our references. Please let us know if any additional adjustments are needed.

“What were the STATA commands used - please specify clearly?”

**Authors:** We appreciate the opportunity to clarify the STATA commands used in our analysis. All STATA code used in this study has been carefully documented and organized in separate .do files corresponding to each analysis component. All relevant STATA commands have been provided in the supplementary files submitted with this revision.

The file named “2003dofile” includes the STATA code used to calculate the regression coefficient(negative **β**) for the 31st province in the year 2003. By modifying the province number from 31 to values between 1 and 30, the regression coefficients for the remaining provinces can be obtained. This requires running the code 31 times, once for each province.

Additionally, the STATA codes used to generate Figures 2 to 7 are also included in their respective dofile files. We hope this supplementary material addresses your concern and allows for full reproducibility of our results.

“= frequency of the word in the corpus” Did you use raw count of words; or the percentage?

**Authors:** Thank you for your careful reading and constructive comment regarding the measurement of word frequency in our study. In this study, absolute raw word counts were used to calculate word frequencies, not percentages.

Line 356: DELETE “contradicting Naranan and Balasubrahmanyan’s claim [37].” unnecessarily contentious as I am not sure we can go into the claims and debae with the limited information in this paper.

**Authors:** We agree and have removed the phrase.

Table 2: use only appropriate number of decimals - to make it easier for the readers.

**Authors:** Thank you for your valuable suggestion regarding the presentation of numerical values in Table 2. We have carefully revised the table to improve its readability, reducing excessive decimal places to 3.

Please ensure that you refer to Table 2 in your text as, if accepted, production will need this reference to link the reader to the Table.

**Authors:** Thank you for this helpful reminder. We have now carefully checked the manuscript and ensured that Table 2 is explicitly referenced in the text where appropriate (see lines 327-330 in the revised manuscript). We appreciate your attention to this detail, which will improve the clarity and navigability of the paper for readers.

We believe these revisions address all the points raised and have further strengthened the manuscript. We are grateful for the valuable suggestions provided, and we hope the revised manuscript now meets the expectations for publication.

Thank you again for your time and consideration.

**Specific Responses to Reviewer Comments:**

While revising your submission, please upload your figure files to the Preflight Analysis and Conversion Engine (PACE) digital diagnostic tool, https://pacev2.apexcovantage.com/. PACE helps ensure that figures meet PLOS requirements. To use PACE, you must first register as a user. Registration is free. Then, login and navigate to the UPLOAD tab, where you will find detailed instructions on how to use the tool. If you encounter any issues or have any questions when using PACE, please email PLOS at figures@plos.org. Please note that Supporting Information files do not need this step.

**Authors:** Thank you for your guidance regarding figure file submission. We confirm that all figures (Figs1-7) have been successfully uploaded and validated through the PACE system, fully complying with PLOS ONE’s technical requirements for resolution, format, and accessibility standards. Should any additional adjustments be needed, we remain available to address them promptly.
