# Supplementary material for: Zipf’s law in China’s local government work reports: A 21-year study using natural language processing and regression analysis
Source: PLoS One. 2025 May 20;20(5):e0324713. doi: 10.1371/journal.pone.0324713 (PMC12091883; doi:10.1371/journal.pone.0324713)
Supplement: Attachment — Submitted filename: response_letter_auresp_5.docx [file pone.0324713.s010.docx]

Dear Dr. Sundar Balakrishnan,

Thank you for your thoughtful feedback. We sincerely appreciate your time and expertise in reviewing our manuscript. Below are our responses and revisions addressing your concerns. The editor’s original comments are marked in blue, with our responses in black.

Lognormal vs. Power-Law Terminology

The phrase “based on the analysis of lognormal distributions” is misleading. Zipf’s Law implies a power-law distribution, not a lognormal.

**Authors:** We fully acknowledge that Zipf’s Law implies a power-law distribution, not a lognormal distribution. In our methodology, we applied a logarithmic transformation to both sides of the power-law relationship to linearize the model for regression analysis. This transformation is a standard approach to empirically test Zipf’s Law while preserving its theoretical power-law foundation. We have now removed any potentially misleading references to lognormal distributions.

We appreciate this opportunity to correct the terminology and better align our presentation with the theoretical foundations of Zipf’s Law.

Regression Specification – Estimated vs. Fixed Slope

Your current equation:

log(S_it) = log(c) - log(R_it) + ε

is not what I suspect you estimate! As implies a fixed slope of –1, which assumes perfect adherence to Zipf’s Law. If the goal is to test Zipf’s Law empirically, I would recommend estimating the slope from the data instead of fixing it. The more general and commonly used formulation is:

log(S_it) = β_0 + β_1 * log(R_it) + ε

Then, you can test whether β_1 ≈ –1 to assess the degree of adherence to Zipf’s Law.

**Authors:** We sincerely apologize for the oversight in our initial equation presentation—the coefficient *β* was inadvertently omitted during typesetting.

The corrected model should indeed include *β* as follows:

$\log S_{it}=l\mathrm{og} c-\beta\log R_{it}+\varepsilon$,

Here $l\mathrm{og}c$ is equivalent to *β_0* in your notation, and $-\beta$ corresponds to *β_1*.We have retained the *β* notation because our subsequent analysis directly interprets *β* rather than *β_1*. We appreciate your attention to this detail and hope the updated version addresses your concern. Please let us know if further modifications are needed.

IMPORTANT: Please read the paper over to double check for any and all small errors and edits.

**Authors:** As requested, we have carefully proofread the entire manuscript multiple times to address minor errors and editorial issues. All revisions are highlighted in red text in the revised manuscript for your convenience. Key modifications include:

1. The misspelled “Guandong Province” in Table 1 and 2 has been corrected to “Guangdong Province” .
2. Mathematical symbols (e.g., *β, S, R*) are now consistently italicized.

Equations are formatted to align with journal guidelines (e.g., centered display, proper spacing).

3. Statistical descriptors in Table 1 and Table 2 (e.g., “Std. dev.,” “Min*,” “Max*”) have been standardized to ensure clarity and consistency.
4. The term “centrally-governed” has been revised to “centrally governed” to adhere to grammatical conventions.
5. All instances of year ranges (e.g., “2003-2023”) and all page range separators in the reference list (e.g., “249-257”) have been updated to use the correct en dash (e.g., “2003–2023”, “249–257” ) for consistency with academic formatting standards.
6. The lowercase “r” in line 242 (originally referencing the variable R) has been revised to uppercase “R” to align with the standardized notation for rank order in mathematical formulas.

Please ensure that you refer to Figure 1 to 7 in your text as, if accepted, production will need this reference to link the reader to the figure.

Authors: Thank you for your careful review. I have thoroughly checked the manuscript as suggested and confirmed that Figures 1 to 7 are explicitly cited in the main text.

These revisions enhance the manuscript’s precision, readability, and adherence to academic standards. We sincerely appreciate your attention to details and we hope the revised manuscript now meets the expectations for publication.

Thank you again for your time and consideration.
