# Supplementary material for: Zipf’s law in China’s local government work reports: A 21-year study using natural language processing and regression analysis
Source: PLoS One. 2025 May 20;20(5):e0324713. doi: 10.1371/journal.pone.0324713 (PMC12091883; doi:10.1371/journal.pone.0324713)
Supplement: Attachment — Submitted filename: response_letter_auresp_6.docx [file pone.0324713.s011.docx]

Dear Dr. Sundar Balakrishnan,

Thank you for your thoughtful feedback. We sincerely appreciate your time and expertise in reviewing our manuscript. Below are our responses and revisions addressing your concerns. The editor’s original comments are marked in blue, with our responses in black.

Line 249 Correction:

Currently: “ = 1,2 … 31 ,t”

Correction Needed: Insert a semicolon after “31” and add a space before “t”: “ = 1,2 … 31; t”

**Authors:** We sincerely appreciate your attention to detail. Regarding the correction noted for Line 249, We confirm that this correction has been made in the revised manuscript.

Lines 307–308 Restatement:

Current statement: "...only 1% of the coefficients fall below 0.722, while 10% are below 0.791. This suggests that the content distribution in most local government work reports exhibits a high degree of concentration, ..."

Suggested revision: Clarify this statement in alignment with your definition provided in lines 234–237. Given your definition that a coefficient (β) greater than 1 indicates a more skewed distribution dominated by high-frequency words, and a coefficient less than 1 indicates a flatter, more even distribution, it would be clearer to explicitly state that none of the coefficients exceed 1, indicating overall a relatively even distribution of word frequencies.

**Authors:** We agree with your suggestion to better align our interpretation with the definition of the Zipf coefficient (*β*) provided in Section 3.2 (Lines 234–237). In the revised manuscript, we have modified the statement to read:

“... only 1% of the coefficients fall below 0.722, while 10% are below 0.791. With all *β* values collectively below 1 (indicating a flatter, more even distribution of word frequencies, as defined in Section 3.2), these results demonstrate that word frequency distributions in local government work reports exhibit relative balance, with minimal skewness toward high-frequency dominance.”

We believe this change better reflects our findings while maintaining consistency with the theoretical framework.

Thank you again for your careful review. Please let us know if any further revisions are required. We appreciate your time and consideration.
